# Association between Reduced Serum Zinc and Diastolic Dysfunction in Maintenance Hemodialysis Patients

**DOI:** 10.3390/nu13062077

**Published:** 2021-06-17

**Authors:** Jiun-Chi Huang, Ya-Chin Huang, Pei-Yu Wu, Wen-Hsien Lee, Yi-Chun Tsai, Yi-Ping Chen, Szu-Chia Chen, Ho-Ming Su, Yi-Wen Chiu, Jer-Ming Chang

**Affiliations:** 1 Division of Nephrology, Department of Internal Medicine, Kaohsiung Medical University Hospital, Kaohsiung Medical University, Kaohsiung 807, Taiwan; karajan77@gmail.com (J.-C.H.); wpuw17@gmail.com (P.-Y.W.); lidam65@yahoo.com.tw (Y.-C.T.); chiuyiwen@gmail.com (Y.-W.C.); jemich@cc.kmu.edu.tw (J.-M.C.); 2 Department of Internal Medicine, Kaohsiung Municipal Siaogang Hospital, Kaohsiung Medical University, Kaohsiung 812, Taiwan; cooky-kmu@yahoo.com.tw; 3 Faculty of Medicine, College of Medicine, Kaohsiung Medical University, Kaohsiung 807, Taiwan; 4 Department of Preventive Medicine, Kaohsiung Municipal Ta-Tung Hospital, Kaohsiung Medical University, Kaohsiung 801, Taiwan; jasimine0603@gmail.com; 5 Department of Occupational & Environmental Medicine, Kaohsiung Medical University Hospital, Kaohsiung Medical University, Kaohsiung 807, Taiwan; 6 Division of Cardiology, Department of Internal Medicine, Kaohsiung Medical University Hospital, Kaohsiung 807, Taiwan; 7 Department of Laboratory Medicine, Kaohsiung Municipal Siaogang Hospital, Kaohsiung 812, Taiwan; 80475@kmuh.org.tw

**Keywords:** serum zinc, diastolic function, E/e’ ratio, left atrial volume index, hemodialysis

## Abstract

Diastolic dysfunction is an emerging challenge among hemodialysis (HD) patients, and the associations between serum zinc with echocardiographic parameters and diastolic function remain uncertain. A total of 185 maintenance HD patients were stratified by the tertiles of serum zinc level to compare their clinical characteristics and echocardiography. Correlations of serum zinc levels with echocardiographic parameters were examined using Pearson’s analysis. Univariate and multivariate logistic regression analyses were performed to investigate the determinants of E/e’ ratio >15 and left atrial volume index (LAVI) > 34 mL/m^2^, both indicators of diastolic dysfunction. Patients belonging to the first tertile of serum zinc level had a significantly higher E/e’ ratio and LAVI. Serum zinc levels were negatively correlated with E (*r* = −0.204, *p* = 0.005), E/e’ ratio (*r* = −0.217, *p* = 0.003), and LAVI (*r* = −0.197, *p* = 0.007). In a multivariate analysis, older age, diabetes, coronary artery disease, and lower serum zinc levels (OR = 0.974, 95% CI = 0.950–0.999, *p* = 0.039) were significantly associated with E/e’ ratio >15. Furthermore, diabetes and lower serum zinc levels (OR = 0.978, 95% CI = 0.958–0.999, *p* = 0.041) were significantly associated with LAVI >34 mL/m^2^. Reduced serum zinc level was significantly associated with diastolic dysfunction among HD patients. Further prospective studies are warranted to investigate whether zinc supplementation can attenuate cardiac dysfunction in maintenance HD patients.

## 1. Introduction

The prevalence of chronic kidney disease (CKD) has increased rapidly in the past two decades [1], and it currently affects about one in ten adults worldwide [2,3]. Approximately 697.5 million individuals have CKD [1], and more than 2.6 million patients require maintenance dialysis globally; this number is estimated more than double by 2030 [4], becoming a huge burden on healthcare systems. In particular, heart failure is a common complication and major cause of cardiovascular mortality in patients on dialysis [5]. Around 87% of patients with end-stage renal disease (ESRD) have major structural abnormalities on echocardiography, of which left atrial (LA) dilation, left ventricular (LV) hypertrophy, and LV diastolic impairment are the most common findings [6]. Diastolic dysfunction is associated with adverse outcomes and is an underemphasized challenge in hemodialysis (HD) patients [7]. The early mitral inflow diastolic velocity (E) to average septal and lateral early diastolic mitral annular velocities (e’) ratio, as well as left atrial volume index (LAVI), are important indicators of diastolic dysfunction [8], and can reflect LV filling pressure; thus, they have prognostic implications in dialysis patients [9,10].

Zinc is an essential microelement with crucial biological functions, and it participates in various roles of tissue repair, immune cell function, cell growth, and antioxidant and anti-inflammatory effects in humans [11,12,13]. Up to 40–78% of patients undergoing HD have been reported to have a zinc deficiency [14,15]. The effects of pharmacological strategies targeting diastolic dysfunction in HD patients remain unsatisfactory [16]; however, accumulating evidence indicates that zinc plays a pivotal role in attenuating cardiac remodeling through the modulation of antioxidant enzymes [17,18]. Furthermore, a recent meta-analysis of 27 case–control studies reported significantly lower serum zinc levels in patients with heart failure than in control subjects [19]. Nevertheless, the associations between serum zinc level with echocardiographic parameters and cardiac function in HD patients are unclear. Therefore, this study aimed to investigate the relationships between serum zinc level and important parameters of systolic and diastolic functions on echocardiography in patients undergoing HD.

## 2. Materials and Methods

### 2.1. Study Participants

**Figure 1:**
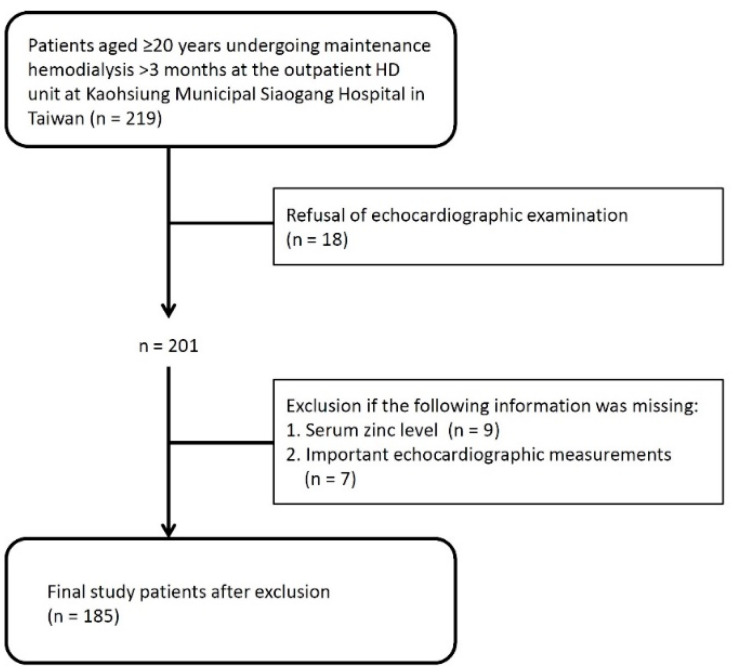
Study population and flowchart.

All patients aged ≥20 years undergoing maintenance HD thrice weekly for >3 months at the outpatient HD unit at Kaohsiung Municipal Siaogang Hospital in Taiwan (n = 219) were screened and enrolled in this study. Each HD treatment was conducted for 3.5–4 h and used a dialyzer with blood flow rates ranging 250–350 mL/min and a dialysate flow rate of 500 mL/min. Eighteen patients refused to receive echocardiographic examinations on study enrollment. In addition, patients who lacked serum zinc measurement (n = 9) or important echocardiographic measurements (n = 7) were also excluded from this study. Finally, 185 maintenance HD patients were included (Figure 1). The study protocol (KMUHIRB-E(I)-20170200) was approved by the Institutional Review Board of Kaohsiung Medical University Hospital. All study patients provided written informed consent. The methods were carried out in accordance with the approved guidelines.

### 2.2. Demographic, Medical and Biochemical Information

Data on the study patients’ demographic information, including age, gender, HD vintage, smoking habits, and comorbidities, were obtained in face-to-face interviews with physicians and from their medical records. Body mass index was calculated as the weight in kilograms divided by the square of height in meters. Blood pressure was measured using standard sphygmomanometers while seated after resting for at least three minutes. Overnight fasting blood samples about 10 mL were obtained before HD sessions for biochemical measurements. The samples were centrifuged at 3500 rpm for 6 min and refrigerated at 2–8 °C after serum isolation. Serum albumin, total cholesterol, high density lipoprotein cholesterol (HDL-C), low density lipoprotein cholesterol (LDL-C), triglycerides (TG), total calcium, phosphorous, potassium, and high sensitivity C-reactive protein (hs-CRP) were measured using an automated chemistry analyzer TBA-c16000 (Toshiba, Tokyo, Japan). An intact parathyroid hormone (iPTH) assay was performed based on a chemiluminescent microparticle immunoassay using the analyzer Immulite 2000 (Siemens Healthcare Diagnostics, Munich, Germany) [20]. Hemoglobin levels were measured using an automated hematology analyzer XN-10 (Sysmex Corporation, Kobe, Japan). All the analyses conducted at the medical laboratory were ISO 15189 accredited. The capability of tests was monitored through regular routine daily internal quality control testing and external proficiency test. Single-pool Kt/V was determined using the Daugirdas method and was taken to represent dialysis efficiency and adequacy [21]. All the above-mentioned information and data are presented in Table 1.

### 2.3. Measurement of Serum Zinc Levels

Serum zinc levels were determined performed using the flame atomic absorption spectrophotometry, FAAS (AAnalyst 800 PerkinElmer, Waltham, MA, USA). All the reagents used were chemically pure, and analytical reagent grades were used without further purification. The zinc standard solution using for calibration was traceable to the National Institute of Standard Technology SRM 3168a. The settings of FAAS were as follows: wavelength, 213.9 nm; slit width, 0.7 nm; and lamp current, 15 mA. The serum sample (0.4 mL) was diluted 1:3 with deionized water. The limit of quantification was 8.2 μg/dL. The recovery of serum zinc was 97.1% at 60 μg/dL, 101.2% at 109.7 μg/dL, and 104.1% at 161.7 μg/dL, respectively. The quality control used in this method was Seronorm™ Trace Elements Serum Control Level 2 (SERO AS, Billingstad, Norway).

### 2.4. Echocardiographic Measurements

An experienced cardiologist performed the echocardiographic examinations using a Vivid 7 ultrasound system (GE Vingmed, Horten, Norway) with the patient in the left decubitus position on nondialysis days. The E/e’ ratio was determined as early mitral inflow diastolic velocity and average of septal and lateral early diastolic mitral annular velocities using Doppler tissue imaging. The LV ejection fraction (LVEF) was determined using the biplane Simpson’s method, and the LV mass was calculated according to the Devereux formula [22]. The LV mass index (LVMI) was calculated as LV mass divided by body surface area. E-wave deceleration time (EDT) was defined as the time from the peak of the E-wave to the end of early mitral flow. LA volume was calculated using the biplane area-length method. LAVI was defined as left atrial volume divided by body surface area. Relative wall thickness was calculated as (2 × posterior wall thickness in diastole)/LV diastolic diameter. All volumetric measurements on echocardiography were performed in accordance with recommendations of the American Society of Echocardiography and the European Association of Echocardiography [22].

### 2.5. Statistical Analyses

Data are presented as percentages for categorical variables, mean ± standard deviation for continuous variables with approximately normal distribution, or median with an interquartile range for continuous variables with skewed distribution, including dialysis vintage, TG, iPTH, and hs-CRP levels. The study patients were classified by tertiles of serum zinc level (<98.4 μg/dL, 98.4–111.2 μg/dL, and >111.2 μg/dL). Differences in the clinical characteristics among these three patient groups were analyzed by using one-way analysis of variance, followed by the post hoc Bonferroni test. Furthermore, Pearson’s correlation analysis was used to evaluate the relationships between serum zinc levels and echocardiographic parameters. To investigate the determinant factors of the E/e’ ratio > 15 and LAVI > 34 mL/m^2^, indices of diastolic dysfunction [8,23] and univariate and multivariate-adjusted logistic regression analyses were performed. Covariates were included into multivariate binary logistic regression analysis based on their statistical significance in univariate analysis. The statistical analyses were carried out using SPSS version 22.0 (SPSS Inc., Chicago, IL, USA) for Windows. A *p*-value of < 0.05 was considered statistically significant.

## 3. Results

A total of 185 maintenance HD patients (91 men and 94 women, mean age 60.9 ± 11.8 years) with a median HD vintage of 6.8 (interquartile range, 2.5–11.3) years were included in this study. The mean level of serum zinc was 105.5 ± 15.2 μg/dL. Table 1 lists the baseline characteristics of the study patients stratified by the tertile of serum zinc level (<98.4 μg/dL, 98.4–111.2 μg/dL, and >111.2 μg/dL). Patients belonging to the first tertile of serum zinc level were older, had higher prevalence rates of coronary artery disease, congestive heart failure and stroke, higher E value, and higher LA diameter on echocardiography. However, these differences did not achieve statistical significance. Furthermore, the patients belonging to the first tertile of serum zinc level had a significantly lower level of serum albumin, a significantly higher E/e’ ratio, and higher LAVI compared to the patients in the third tertile of serum zinc level.

Table 2 shows the correlations between serum zinc level and echocardiographic parameters. Serum zinc level was significantly negatively correlated with E (*r* = −0.204, *p* = 0.005), E/e’ ratio (*r* = −0.217, *p* = 0.003), and LAVI (*r* = −0.197, *p* = 0.007). However, serum zinc level was not correlated with LVEF, LV mass, LVMI, relative wall thickness, e’, EDT, or LA diameter.

Univariate and multivariate logistic regression analyses were performed to identify the determinant factors associated with E/e’ ratio > 15 and LAVI > 34 mL/m^2^, two important indicators of diastolic dysfunction. In univariate analysis, older age, diabetes mellitus, coronary artery disease, and lower serum zinc level were significantly associated with E/e’ ratio > 15. Older age (per 1 year; odds ratio (OR), 1.038; 95% confidence interval (CI), 1.004–1.074; *p* = 0.029), diabetes mellitus (OR, 2.559; 95% CI, 1.249–5.244; *p* = 0.010), coronary artery disease (OR, 2.569; 95% CI, 1.012–6.517; *p* = 0.047), and lower serum zinc level (per 1 μg/dL, OR, 0.974; 95% CI, 0.950–0.999; *p* = 0.039) were significantly associated with E/e’ ratio > 15 in the multivariate analysis (Table 3).

Table 4 shows the determinant factors for LAVI > 34 mL/m^2^. In univariate analysis, diabetes mellitus, lower total cholesterol, lower LDL-C levels, and lower serum zinc level were significantly associated with LAVI > 34 mL/m^2^. Diabetes mellitus (OR, 2.453; 95% CI, 1.312–4.585; *p* = 0.005) and lower serum zinc level (per 1 μg/dL, OR, 0.978; 95% CI, 0.958–0.999; *p* = 0.041) were significantly associated with LAVI > 34 mL/m^2^ in the multivariate analysis.

## 4. Discussion

In this study, we did not find significant differences in most clinical characteristics among the HD patients in the three tertiles of serum zinc level, except for a significantly lower serum albumin level, significantly higher E/e’ ratio, and higher LAVI in the patients in the first tertile of serum zinc level. In addition, serum zinc level was significantly negatively correlated with E, E/e’ ratio, and LAVI (indices of diastolic function). However, there were no correlations between serum zinc and LVEF (indicator of systolic function), LV mass, and LVMI (indicators of LV hypertrophy). In particular, a lower serum zinc level was significantly associated with the markers of diastolic dysfunction (E/e’ ratio > 15 and LAVI > 34 mL/m^2^).

Indicators of diastolic dysfunction, such as E/e’ ratio and LAVI, have been shown to independently predict ominous prognosis in HD patients [24,25]. The risk factors and their involvement in the pathophysiology of diastolic dysfunction in HD patient populations are complex. In addition to traditional risk factors, such as older age, diabetes, and higher blood pressure, certain uremia-related predisposing factors, such as oxidative stress, malnutrition, inflammation, and uremic toxins, have also been shown to contribute to abnormal myocardial relaxation and ventricular stiffness [16,26]. As this condition progresses, cardiac remodeling and fibrosis lead to increased LV filling pressure and diastolic dysfunction [5,8]. The role of serum zinc in heart failure is unclear due to conflicting results and small sample sizes in previous studies [27,28]. A recent meta-analysis reported that patients with idiopathic dilated cardiomyopathy had significantly reduced serum zinc levels, but this was not found in patients with ischemic cardiomyopathy [19]. In the current study, the patients in the first tertile of serum zinc level had a higher prevalence of congestive heart failure than those in the second and third tertiles of serum zinc level. However, these differences did not achieve statistical significance. This suggests that HD patients with a reduced serum zinc level might have a higher prevalence of heart failure.

We also found that serum zinc levels were negatively correlated with E/e’ ratio and LAVI, but they were not correlated with LVEF, LV mass, or LVMI. These findings are consistent with a previous study by Alexanian et al., who reported that serum zinc levels were significantly negatively correlated with E/e’ ratio but not with LVEF in patients with heart failure [29]. However, the results of this study are different to a report by Huang et al. which reported that serum zinc levels were significantly inversely correlated with LV mass and LVMI [30]. This may be because Huang et al. excluded patients with renal dysfunction, and the mechanisms contributing to LV hypertrophy and systolic dysfunction are more complex in patients with ESRD [31,32].

Another important finding of this study is that lower serum levels were significantly associated with E/e’ ratio > 15 and LAVI > 34 mL/m^2^, indicating that serum zinc may play a role in diastolic dysfunction in HD patients. Recent evidence has suggested several plausible explanations for the link between lower serum zinc levels and diastolic dysfunction. First, serum zinc levels have been associated with fluid volume and nutritional status in HD patients [33]. Taste changes are common and associated with malnutrition in maintenance dialysis patients [34], and they may result in decreased intake of calories and zinc-rich foods. Second, lower serum zinc has been associated with intradialytic hypertension in HD patients [35]. Zinc has been shown to be involved in the regulation of arterial pressure [36], and zinc deficiency may impair downstream actions of endothelial nitric oxide, leading to insufficient vasodilation [37]. Moreover, a reduced serum zinc level could be harmful to antioxidant defenses, leading to oxidative stress-induced cardiomyocyte necrosis [38]. In an animal study, zinc was shown to attenuate cardiac remodeling through the modulation of antioxidant enzymes [17]. In addition, a randomized control trial evaluating nutritional supplementation, including zinc, in patients with heart failure found an improvement in LV end-diastolic volume after treatment [39]. Taken together, the role of zinc may be underestimated among HD patients, and further studies are needed to explore the pathways linking reduced serum levels to diastolic dysfunction.

There are several limitations to this study. First, the sample size of study patients was relatively small. In addition, although certain echocardiographic parameters are volume dependent, echocardiography was performed on nondialysis days and when patients were close to their dry weight to minimize the influences of hypervolemia. Moreover, a cross-sectional study design can only evaluate the associations between serum zinc levels and diastolic function of the heart in HD patients, and we did not have information on the extra supplementation of micronutrients among the study patients. Future large-scale prospective studies are needed to investigate the causal relationship between serum zinc and diastolic function, and to determine whether zinc supplementation is beneficial for diastolic dysfunction in maintenance HD patients.

## 5. Conclusions

This study highlights the significant association between a reduced serum zinc level and diastolic dysfunction in patients on HD. Serum zinc level was significantly correlated with the parameters of diastolic function, but was not correlated with indicators of LV hypertrophy and systolic function. Future prospective studies are necessary to investigate whether zinc supplementation can attenuate cardiac dysfunction in maintenance HD patients.

## Figures and Tables

**Table 1:** Comparison of baseline characteristics among study patients classified by tertiles of serum zinc level.

Characteristics	1st Tertile of Serum Zinc (<98.4 μg/dL) (n = 62)	2nd Tertile of Serum Zinc (98.4–111.2 μg/dL) (n = 62)	3rd Tertile of Serum Zinc (>111.2 μg/dL) (n = 61)	*p*-Value
Age (year)	62.9 ± 12.2	60.9 ± 10.2	58.9 ± 12.7	0.184
Men (%)	43.5	51.6	57.4	0.305
Dialysis vintage (year)	6.5 (1.4–12.4)	6.1 (2.6–10.7)	8.8 (3.7–11.9)	0.232
Diabetes mellitus (%)	48.4	50.0	39.3	0.443
Coronary artery disease (%)	19.4	9.7	14.8	0.312
Congestive heart failure (%)	33.9	22.6	19.7	0.160
Stroke (%)	12.9	9.7	6.6	0.494
Current smoking (%)	9.7	14.5	16.4	0.531
Body mass index (kg/m^2^)	23.4 ± 3.6	23.5 ± 3.2	23.8 ± 4.2	0.808
Systolic BP (mmHg)	148.8 ± 24.5	162.8 ± 28.6 *	156.1 ± 28.8	0.030
Diastolic BP (mmHg)	77.9 ± 15.3	86.5 ± 14.0 *	81.2 ± 17.2	0.016
**Laboratory parameters**				
Albumin (g/dL)	3.75 ± 0.29	3.85 ± 0.36	3.94 ± 0.29 *	0.005
Hemoglobin (g/dL)	10.4 ± 1.1	10.4 ± 1.3	10.7 ± 1.3	0.270
Total cholesterol (mg/dL)	177.3 ± 40.6	180.0 ± 44.8	178.9 ± 35.5	0.897
HDL-C (mg/dL)	41.8 ± 12.1	40.5 ± 12.7	42.1 ± 12.1	0.748
LDL-C (mg/dL)	83.8 ± 29.3	89.4 ± 30.8	83.1 ± 24.8	0.406
Triglycerides (mg/dL)	123.5 (90.5–206)	147.5(79.8–206.8)	117.0 (83.5–233.5)	0.674
Total calcium (mg/dL)	9.2 ± 1.1	9.3 ± 1.1	9.2 ± 0.9	0.607
Phosphorous (mg/dL)	4.6 ± 1.4	4.4 ± 0.9	4.5 ± 1.1	0.508
Potassium (mmol/L)	4.6 ± 0.8	4.7 ± 0.7	4.6 ± 0.7	0.836
iPTH (pg/mL)	442 (226–733)	342 (151–559)	335 (164–552)	0.123
hs-CRP (mg/L)	3.2 (1.4–7.0)	1.7 (0.8–4.6)	2.2 (1.0–8.6)	0.773
spKt/V	1.6 ± 0.3	1.6 ± 0.3	1.6 ± 0.3	0.778
Zinc (μg/dL)	89.4 ± 6.7	104.8 ± 3.5 *	122.5 ± 9.2 *^,^^†^	<0.001
**Echocardiographic parameters**				
LVEF (%)	65.7 ± 9.8	66.0 ± 10.1	68.4 ± 9.9	0.256
LV mass (g)	218.7 ± 74.4	229.7 ± 82.3	221.8 ± 73.0	0.712
LVMI (g/m2)	133.6 ± 38.7	140.5 ± 49.1	133.3 ± 39.5	0.566
Relative wall thickness	0.42 ± 0.11	0.41 ± 0.10	0.43 ± 0.10	0.576
E (cm/s)	89.6 ± 37.5	81.4 ± 28.4	77.4 ± 22.9	0.075
e’ (cm/s)	6.5 ± 2.4	6.6 ± 2.4	7.0 ± 2.1	0.441
EDT (ms)	179.2 ± 55.8	193.4 ± 71.7	193.9 ± 57.3	0.328
E/e’ ratio	16.7 ± 1.3	14.1 ± 0.8	12.3 ± 0.6 *	0.034
LA diameter (cm)	3.8 ± 0.6	3.5 ± 0.6	3.5 ± 0.8	0.051
LAVI (mL/m^2^)	37.2 ± 12.3	32.3 ± 11.5	30.8 ± 10.3 *	0.005

Abbreviations: BP, blood pressure; HDL-C, high density lipoprotein cholesterol; LDL-C, low density lipoprotein cholesterol; iPTH, intact parathyroid hormone; hs-CRP, high sensitivity C-reaction protein; spKt/V: single-pool Kt/V; LVEF, left ventricular ejection fraction; LV, left ventricular; LVMI, left ventricular mass index; E, early mitral inflow diastolic velocity; e’, average of septal and lateral early diastolic mitral annular velocities; EDT, E-wave deceleration time; LA, left atrial; LAVI, left atrial volume index. * p < 0.05 compared with the 1st tertile of serum zinc level; ^†^ *p* < 0.05 compared with the 2nd tertile of serum zinc level.

**Table 2:** Correlation between serum zinc level and echocardiographic parameters in study patients.

Echocardiographic Parameters	Pearson’s *r*	*p*-Value
LVEF (%)	0.072	0.332
LV mass (g)	−0.005	0.945
LVMI (g/m^2^)	−0.057	0.438
Relative wall thickness	0.082	0.269
E (cm/s)	−0.204	0.005
e’ (cm/s)	0.112	0.131
EDT (ms)	0.131	0.076
E/e’ ratio	−0.217	0.003
LA diameter (cm)	−0.134	0.069
LAVI (mL/m^2^)	−0.197	0.007

Abbreviations: LVEF, left ventricular ejection fraction; LV, left ventricular; LVMI, left ventricular mass index; E, early mitral inflow diastolic velocity; e’, average of septal and lateral early diastolic mitral annular velocities; EDT, E-wave deceleration time; LA, left atrial; LAVI, left atrial volume index.

**Table 3:** Determinant factors of E/e’ ratio > 15 using univariate and multivariate logistic regression.

Covariates	Univariate		Multivariate	
	OR (95% CI)	*p*-Value	OR (95% CI)	*p*-Value
Age (per 1 year)	1.056 (1.024–1.089)	0.001	1.038 (1.004–1.074)	0.029
Sex (male vs. female)	1.237 (0.658–2.329)	0.509	–	–
Current smoking	1.131 (0.457–2.801)	0.790	–	–
Diabetes mellitus	3.512 (1.803–6.838)	<0.001	2.559 (1.249–5.244)	0.010
Coronary artery disease	4.438 (1.900–10.369)	0.001	2.569 (1.012–6.517)	0.047
Stroke	2.043 (0.760–5.491)	0.157	–	–
Dialysis vintage (per 1 year)	0.979 (0.923–1.039)	0.482	–	–
Body mass index (per 1 kg/m^2^)	0.971 (0.889–1.061)	0.513	–	–
Systolic BP (per 1 mmHg)	1.011 (0.998–1.024)	0.098	–	–
Diastolic BP (per 1 mmHg)	0.990 (0.968–1.012)	0.383	–	–
Albumin (per 1 g/dL)	0.475 (0.179–1.258)	0.134	–	–
Hemoglobin (per 1 g/dL)	0.961 (0.744–1.241)	0.760	–	–
Total cholesterol (per 1 mg/dL)	0.995 (0.987–1.003)	0.217	–	–
Triglycerides (per log mg/dL)	1.296 (0.402–4.172)	0.664	–	–
HDL-C (per 1 mg/dL)	0.988 (0.962–1.015)	0.381	–	–
LDL-C (per 1 mg/dL)	0.998 (0.987–1.010)	0.777	–	–
Phosphate (per 1 mg/dL)	0.947 (0.718–1.250)	0.701	–	–
Total calcium (per 1 mg/dL)	1.221 (0.900–1.657)	0.199	–	–
Potassium (per 1 mmol/L)	0.888 (0.576–1.369)	0.591	–	–
iPTH (per log pg/mL)	1.388 (0.641–3.007)	0.406	–	–
hs-CRP (per log mg/L)	1.679 (0.969–2.908)	0.065	–	–
spKt/V (per 1 unit)	1.037 (0.356–3.021)	0.947	–	–
Serum zinc (per 1 μg/dL)	0.970 (0.948–0.992)	0.008	0.974 (0.950–0.999)	0.039

Abbreviations: BP, blood pressure; HDL-C, high density lipoprotein cholesterol; LDL-C, low density lipoprotein cholesterol; iPTH, intact parathyroid hormone; hs-CRP, high sensitivity C-reaction protein; spKt/V: single-pool Kt/V.

**Table 4:** Determinant factors of LAVI > 34 mL/m^2^ using univariate and multivariate logistic regression.

Covariates	Univariate		Multivariate	
	OR (95% CI)	*p*-Value	OR (95% CI)	*p*-Value
Age (per 1 year)	1.009 (0.984–1.035)	0.468	–	–
Sex (male vs. female)	1.366 (0.754–2.473)	0.303	–	–
Current smoking	1.538 (0.659–3.590)	0.319	–	–
Diabetes mellitus	2.507 (1.367–4.595)	0.003	2.453 (1.312–4.585)	0.005
Coronary artery disease	2.215 (0.970–5.057)	0.059	–	–
Stroke	1.287 (0.483–3.433)	0.614	–	–
Dialysis vintage (per 1 year)	0.960 (0.908–1.016)	0.158	–	–
Body mass index (per 1 kg/m^2^)	1.015 (0.936–1.110)	0.719	–	–
Systolic BP (per 1 mmHg)	1.009 (0.998–1.021)	0.117	–	–
Diastolic BP (per 1 mmHg)	1.006 (0.986–1.026)	0.550	–	–
Albumin (per 1 g/dL)	0.891 (0.355–2.237)	0.805	–	–
Hemoglobin (per 1 g/dL)	0.941 (0.740–1.197)	0.622	–	–
Total cholesterol (per 1 mg/dL)	0.992 (0.984–1.000)	0.042	0.998 (0.985–1.010)	0.691
Triglycerides (per log mg/dL)	0.982 (0.328–2.939)	0.975	–	–
HDL-C (per 1 mg/dL)	0.995 (0.971–1.020)	0.686	–	–
LDL-C (per 1 mg/dL)	0.986 (0.975–0.997)	0.015	0.988 (0.971–1.006)	0.196
Phosphate (per 1 mg/dL)	1.094 (0.845–1.145)	0.495	–	–
Total calcium (per 1 mg/dL)	1.083 (0.815–1.439)	0.585	–	–
Potassium (per 1 mmol/L)	1.227 (0.822–1.832)	0.317	–	–
iPTH (per log pg/mL)	1.185 (0.587–2.392)	0.636	–	–
hs-CRP (per log mg/L)	0.875 (0.526–1.456)	0.608	–	–
spKt/V (per 1 unit)	0.576 (0.207–1.600)	0.290	–	–
Serum zinc (per 1 μg/dL)	0.978 (0.958–0.998)	0.034	0.978 (0.958–0.999)	0.041

Abbreviations: BP, blood pressure; HDL-C, high density lipoprotein cholesterol; LDL-C, low density lipoprotein cholesterol; iPTH, intact parathyroid hormone; hs-CRP, high sensitivity C-reaction protein; spKt/V: single-pool Kt/V.

## Data Availability

The data presented in this study are available on request from the corresponding author.
